# Comparative Genome Analysis of the Lignocellulose Degrading Bacteria *Citrobacter freundii* so4 and *Sphingobacterium multivorum* w15

**DOI:** 10.3389/fmicb.2020.00248

**Published:** 2020-03-03

**Authors:** Larisa Cortes-Tolalpa, Yanfang Wang, Joana Falcao Salles, Jan Dirk van Elsas

**Affiliations:** Cluster of Microbial Ecology, Groningen Institute for Evolutionary Life Sciences, University of Groningen, Groningen, Netherlands

**Keywords:** comparative analysis, synergism, lignocellulose, degradation, bacteria

## Abstract

Two bacterial strains, denoted so4 and w15, isolated from wheat straw (WS)-degrading microbial consortia, were found to grow synergistically in media containing WS as the single carbon and energy source. They were identified as *Citrobacter freundii* so4 and *Sphingobacterium multivorum* w15 based on 16S rRNA gene sequencing and comparison to the respective *C. freundii* and *S. multivorum* type strains. In order to identify the mechanisms driving the synergistic interactions, we analyzed the draft genomes of the two strains and further characterized their metabolic potential. The latter analyses revealed that the strains had largely complementary substrate utilization patterns, with only 22 out of 190 compounds shared. The analyses further indicated *C. freundii* so4 to primarily consume amino acids and simple sugars, with laminarin as a key exception. In contrast, *S. multivorum* w15 showed ample capacity to transform complex polysaccharides, including intermediates of starch degradation. Sequence analyses revealed *C. freundii* so4 to have a genome of 4,883,214 bp, with a G + C content of 52.5%, 4,554 protein-encoding genes and 86 RNA genes. *S. multivorum* w15 has a genome of 6,678,278 bp, with a G + C content of 39.7%, 5,999 protein-encoding genes and 76 RNA genes. Genes for motility apparatuses (flagella, chemotaxis) were present in the genome of *C. freundii* so4, but absent from that of *S. multivorum* w15. In the genome of *S. multivorum* w15, 348 genes had regions matching CAZy family enzymes and/or carbohydrate-binding modules (CBMs), with 193 glycosyl hydrolase (GH) and 50 CBM domains. Remarkably, 22 domains matched enzymes of glycoside hydrolase family GH43, suggesting a strong investment in the degradation of arabinoxylan. In contrast, 130 CAZy family genes were found in *C. freundii* so4, with 61 GH and 12 CBM domains identified. Collectively, our results, based on both metabolic potential and genome analyses, revealed the two strains to harbor complementary catabolic armories, with *S. multivorum* w15 primarily attacking the WS hemicellulose and *C. freundii* so4 the cellobiose derived from cellulose, next to emerging oligo- or monosaccharides. Finally, *C. freundii* so4 may secrete secondary metabolites that *S. multivorum* w15 can consume, and detoxify the system by reducing the levels of (toxic) by-products.

## Introduction

Agricultural waste, such as wheat straw (WS), maize straw and sugar cane bagasse constitutes lignocellulosic biomass (LCB), which is composed of three main components: cellulose, hemicellulose and lignin. The proportion of these components is dependent on the plant species, as well as on the time and conditions of growth (Sorek et al., 2014). LCB substrates represent promising alternatives to carbon sources for the production of useful compounds such as plastics or biodiesel (Guerriero et al., 2016). The utilization of LCB is particularly relevant as mankind is threatened by the depletion of sources of energy as well as global warming (Kumar et al., 2015).

The degradation of LCB not only requires a large variety of hydrolytic enzymes, i.e., cellulases, hemicellulases, and ligninases (Himmel et al., 2007), but also, from each of these three enzymes groups, different types of enzymes with different cleavage specificities. For complete degradation, the (additional) action of carbohydrate-binding modules (CBMs), which bind cellulose or hemicelluloses, and helper enzymes such as lytic polysaccharide monooxygenases (LPMOs), xylan esterases (CEs), and polysaccharide lyases (PLs) are necessary (Koeck et al., 2014).

Degradation of LCB is a complex process. In nature, it is only efficient if diverse microorganisms, mainly bacteria and fungi, contribute (Cragg et al., 2015). These organisms produce diverse lytic as well as auxiliary enzymes, which work in a synergistic manner (Lynd et al., 2002). Moreover, depending on the type of substrate, interactions within the degrader microbial communities emerge, which could be either positive or negative.

“Division of labor” (DOL) is one of the strategies used by microorganisms for dealing with complex substrates (Jiménez et al., 2017). DOL is observed – for example – in a microbial food chain when it is necessary to degrade complex organic compounds. There are examples in the cycles of carbon, as well as of sulfur and nitrogen (Falkowski et al., 2008). According to West and Cooper (2016), DOL can be defined as the cooperation between individuals that are each specialized in specific tasks. Some of the requirements for DOL are (1) presence of diverse phenotypes (individuals that perform different tasks), (2) cooperation, in which the tasks performed by one individual will benefit the other individual, and (3) division of tasks favoring adaptation to the environment (increasing the fitness of the individuals involved). The finding of growth synergy was in line with data by Deng and Wang, in which the presence of a complex source of carbon and energy stimulates synergistic interactions and reduces antagonistic ones (Deng and Wang, 2016). Although it is likely that DOL plays an important role during the degradation of LCB by microbial communities, it remains unclear how and to which extent such interactions take place during the degradation process. Clearly, a better understanding of the process will improve the design and utilization of microbial consortia at industrial level (Song et al., 2014).

In a quest to unravel the degradation of LCB by microbial communities, a ‘minimal’ lignocellulose degrader consortium, encompassing strains so4 (identified as *Citrobacter freundii* on the basis of 16S ribosomal RNA gene analysis) and w15 (identified as *Sphingobacterium multivorum*), was constructed and examined (Cortes-Tolalpa et al., 2017). The strains had been recovered from, respectively, soil- and wood-derived consortia grown on WS (Cortes-Tolalpa et al., 2016). Synergism in growth was observed exclusively in cultures with complex carbon and energy sources, i.e., WS as well as synthetic recalcitrant biomass (Cortes-Tolalpa et al., 2017), but not in cultures with glucose. Moreover, strains related to w15 and so4 were found to be very abundant in consortia able to degrade diverse LCB substrates (Jiménez et al., 2014b; Brossi de Lima et al., 2015), suggesting their potential key roles in the degradation.

In order to establish the potential mechanisms driving the synergistic interaction between strains so4 and w15 during the WS degradation, we determined the genetic and metabolic capabilities of these two organisms, in particular with respect to their LCB degrading potential. Specifically, we evaluated the potential functional complementation between *C. freundii* so4 and *S. multivorum* w15 by analyzing their substrate utilization patterns, as well as their genome-encoded functional complements, by placing a focus on relevant LCB hydrolytic capacities.

## Materials and Methods

### Strains and Growth Conditions

Strains so4 and w15, referred to as *C. freundii* so4 and *S. multivorum* w15, were isolated from microbial consortia able to degrade raw WS (Cortes-Tolalpa et al., 2016). Both strains were able to grow in monoculture using raw WS as the sole carbon and energy source (Cortes-Tolalpa et al., 2017). For routine purposes, strains were grown in TY medium (10 g/L tryptone; 5 g/L yeast extract, 5 g/L NaCl; pH 7.2; Sigma-Aldrich, Darmstadt, Germany). The cultures were incubated overnight at 28°C and 180 rpm. Motility and temperature range of growth of each strain were determined as in Supplementary Material.

### BIOLOG Phenotype Testing

BIOLOG phenotype micro arrays were used (96-well GN2 and PM2A plates; Biolog Inc., Hayward, CA, United States) to test the catabolic capabilities of *C. freundii* so4 and *S. multivorum* w15. The arrays consisted of totals of 190 carbon sources, encompassing alcohols, amides, amines, amino acids, carbohydrates, carboxylic acids, esters, fatty acids, and polymers. Single colonies of each strain were picked from tryptic soy agar (TSA) plates on which they were subcultured, to produce overnight cultures in TY media (with shaking at 150 rpm, at 28°C). Homogeneous cell suspensions were made with IF-0a GN/GP inoculation fluid (72101) and diluted to 0.001 OD at 590 nm; in the case of the PM2A plate, the inocula were supplemented with 150 μL of Biolog redox dye mix A (100X). The inocula were kept for 2 h at room temperature and then 150 μL of the suspension was added into each well of the plates. The plates were incubated at 28°C and read at 0, 6, 12, 24, 48, 72, and 84 h using a microtiter plate reader at 590 nm (Holmes et al., 1994). Analyses of the data were performed using the area under the (growth) curve (AUC) as the criterion (Kalai Chelvam et al., 2015).

### Genomic DNA Extraction From Strains

Total genomic DNA was extracted from the liquid and shaken TY cultures of the two strains by using the UltraClean DNA Isolation Kit (MoBio^®^ Laboratories Inc., Carslab, United States), following the instructions of the manufacturer.

### Genome Sequencing and Assembly

Whole-genome sequencing of *C. freundii* so4 and *S. multivorum* w15 was performed using the Illumina NextSeq 500 V2 platform by 150-bp paired-end reads (LGC Genomics Gmbh, Berlin, Germany). Assembly and scaffolding of the sequence data were performed using SPAdes 3.5.0, according to the workflow described by Nurk et al. (2013). For *C. freundii* so4, the final assembly resulted in 49 contigs with an N50 of 282,822 bp. For *S. multivorum* w15, 92 contigs were obtained, at an N50 value of 133,589 bp.

### Genome Annotation

Genome drafts were annotated by the Rapid Annotation Subsystem Technology (RAST) (Aziz et al., 2008).

### Metabolic Pathway Comparison

First, we determined the numbers of distinct reactions per metabolic pathway according to the enzyme commission (EC) number; EC numbers specify enzyme-catalyzed reactions and so enzymes can be diverse, for instance among different organisms. Following this first step, and taking the total number of distinct reactions per metabolic pathway as the 100% value, we calculated the percentage of distinct ECs according to the number of ECs found per strain per pathway. Finally, the functionality of the pathway was assessed by using the metabolic tool comparison in RAST. The notion of likely functioning is given by the presence of genes for all the functional roles that constitute a variant of a pathway (or subsystem) (Overbeek et al., 2014).

### Genome Statistics

Predicted complete genes were translated *in silico* and the resulting data used to probe the Pfam database (Finn et al., 2014) as well as the COG database through the MicroScope platform (Vallenet et al., 2017). Signal-P server 4.1 was used to predict signal peptide regions (Petersen et al., 2011). Transmembrane domains were identified using THMMH server 2.0 (Krogh et al., 2001). OrthoFinder (Emms and Kelly, 2015) was used to identify single-copy genes in the genomes. PlasmidFinder was used to check the genomes for the presence of plasmids (Carattoli et al., 2014).

### Phylogenetic Analyses

RNAmmer was used for identification of the rRNA genes (Lagesen et al., 2007). The 16S rRNA gene sequences (NODE_31_length_1713_cov_124.214_ID_61) of *C. freundii* so4 and (NODE_70_length_5327_cov_127.629_ID_139) of *S. multivorum* w15 were thus used for phylogenetic analyses. The 16S rRNA genes from the type strains of *C. freundii* and *S. multivorum*, as well as from closely related strains, were recovered from the SILVA ribosomal RNA database (Quast et al., 2013) and a phylogenetic tree was constructed using the Neighbor Joining (NJ) method. MEGA v 6.0 software was used to calculate pairwise P-distance values. Bootstrap analysis was performed with 1,000 repetitions.

### Carbohydrate-Degradative Enzymes

Predicted genes were translated and the translates were used to search for carbohydrate-active enzymes (dbCAN); as criteria, we used coverage values above 0.5 and *e*–values < 1e-18 (Yin et al., 2012; Zhang et al., 2018). Classifications of the CAZy families were done automatically by dbCAN. CAZy^1^ and CAZypedia^2^ databases were used when necessary for extra information.

### Genome Accession Numbers

This whole genome sequencing project has been deposited at DDBJ/ENA/GenBank under the accession numbers PHGU00000000 and PHGV00000000. The versions described in this manuscript are PHGU01000000 and PHGV01000000. The strains used in this study have been deposited in the German Collection of Microorganisms and Cell Cultures (DMSZ, Braunschweig, Germany). *C. freundii* so4 is deposited under the number DSM 106340T; *S. multivorum* w15 is in the process of obtaining the accession number from DMSZ.

## Results

### Carbon Source Utilization Profiles

#### *C. freundii* so4

**FIGURE 1:**
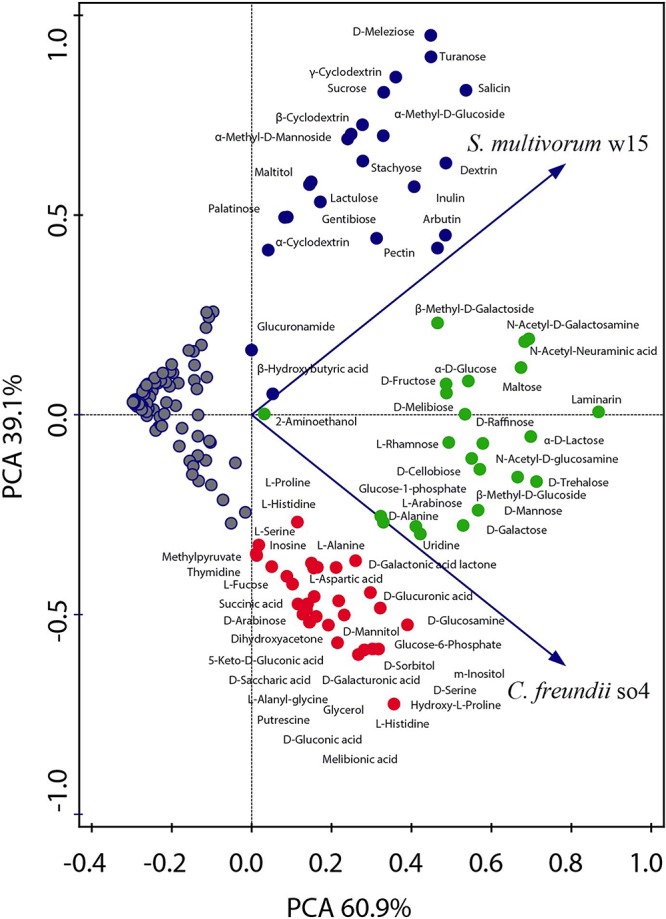
Principal component analysis showing the metabolic capacity of the strains *C. freundii* so4 and *S. multivorum* w15. The ability to consume each of the individual carbon sources was tested using BIOLOG PM2A and GN2 plates. *C. freundii* so4 exhibited the capacity to grow on 52 compounds, mainly intermediate metabolites, amino acids, organic acids, sugar alcohols and monosaccharides (red and green). *S. multivorum* w15 grew on 42 compounds (blue and green), presenting preference for disaccharides, oligosaccharides and polymers. Red symbols indicate the compounds consumed only by *C. freundii* so4; blue symbols those consumed only by *S. multivorum* w15 and green symbols those consumed by both strains.

Overall, *C. freundii* so4 was able to grow on 52 out of the 190 carbon sources tested (Figure 1 and Supplementary Table 1), leaving a total of 138 substrates unused. The organism showed a preference for consumption of simple carbon sources, in particular eight amino acids (L-histidine, hydroxy-L-proline, D-alanine, L-alanine, L-serine, D-serine, L-aspartic acid, and L-alanyl-glycine), eight carboxylic acids (succinic, 5-keto-D-gluconic, D-glucuronic, D, L-lactic, D-galacturonic, D-gluconic, D-saccharic and melibionic acid), four sugar alcohols (glycerol, D-sorbitol, D-mannitol, and *m*-inositol), two monosaccharides (D-arabinose and glucose-6-phosphate), one ketose (dihydroxyacetone) and one deoxy sugar (L-fucose) (Figure 1 and Supplementary Table 1).

#### *S. multivorum* w15

*Sphingobacterium multivorum* w15 could grow on only 42 out of the 190 carbon sources tested (Figure 1 and Supplementary Table 2) leaving 148 substrates unused. Interestingly, *S. multivorum* w15 preferred to consume complex carbon sources, such as five di-saccharides (lactulose, palatinose, sucrose, turanose, and gentibiose), one trisaccharide (D-melezitose, found in honeydew) and one tetrasaccharide (stachyose, found in legume seeds). Furthermore, it revealed an interesting capacity to grow on oligosaccharides derived from plant polymers, i.e., starch (dextrin, α-, β- and γ-cyclodextrins), and polysaccharides such as pectin and inulin (found in plants like chicory) (Figure 1 and Supplementary Table 2).

### Species-Specific and Shared Carbon Sources

Taking the data together, 30 carbon sources were used, as the sole carbon and energy sources, by *C. freundii* so4, but not by *S. multivorum* w15 (Supplementary Table 1). All of these 30 carbon sources are rather simple (small) molecules, with a relatively large share of carboxylic acids (8; 27%). Furthermore, strain so4 can also consume putrescine, which is produced by the breakdown of amino acids in living and dead organisms and is toxic in large doses. Conversely, 20 other compounds were used only by *S. multivorum* w15 (Supplementary Table 2). Most of these are more complex carbohydrates, with eight being compounds from plants, i.e., stachyose, salicin, sucrose, maltitiol, arbutin, inulin, pectin, and dextrin, suggesting a key role of strain w15 in degrading complex LCB.

Regarding shared resources, *C. freundii* so4 and *S. multivorum* w15 were found to consume 22 compounds in similar rates. Nine of these were monosaccharides: α-D-glucose, glucose-1-phosphate, D-fructose, D-mannose, D-galactose, L-arabinose, β-methyl-D-glucose, *N*-acetyl-D-glucosamine and β-methyl-D-galactoside. Five were disaccharides: maltose, D-melibiose, α-D-lactose, D-trehalose, and D-cellobiose. Moreover, D-alanine was the only amino acid that both strains could consume as the single carbon source, whereas the polymer laminarin was also shared between them (Figure 1 and Supplementary Table 3).

### Genome Descriptions

Genomic DNA was successfully isolated and purified from both *C. freundii* so4 and *S. multivorum* w15, and subsequently fully sequenced. The genome sequencing statistics of *C. freundii* so4 and *S. multivorum* w15 can be found in Table 1.

**TABLE 1:** Genome statistics of *C. freundii* so4 and *S. multivorum* w15.

	***C. freundii* so4**		***S. multivorum* w15**	
**Attribute**	**Value**	**% of total**	**Value**	**% of total**
Genome size (bp)	4883214	100	6678278	100
DNA coding region (bp)	4323598	88.54	5967041	89.35
DNA G + C content (bp)	2565641	52.54	2655951	39.77
DNA scaffolds (‘contigs’)	49	–	92	–
Total genes	4703	100	6087	100
Protein-encoding genes	4554	96.83	5999	98.55
RNA genes:	86	1.83	76	1.23
rRNA	11		10	
tRNA	75		66	
Pseudo genes:	–	–	–	–
Genes assigned to COGs	3915	83.25	3854	63.31
Genes assigned Pfam domains	3970	84.41	2871	47.17
Genes codifying signal peptides	416	8.85	691	11.35
Genes coding for transmembrane helices	1106	23.52	1241	20.39
CRISPR repeats	1	–	3	–
Plasmid	–	–	–	–

*–, not detected; rRNA: ribosomal RNA, tRNA: transfer RNA.*

The genome of *Citrobacter freundii* so4, spread over 49 contigs, had a size of 4,883,214 bp, with an average G + C content of 52.5%. Out of the 4,703 annotated genes, 4,554 were protein-encoding genes, of which only 585 were detected as genes encoding hypothetical proteins. A total of 86 RNA-encoding genes was found. Of the latter, 11 encoded ribosomal RNA (nine 5S rRNA, one 23S rRNA, and one 16S rRNA) and 75 tRNA. Only one CRISPR repeat region was found in this genome. Using plasmidfinder, no plasmids were found (Table 1).

The genome of *Sphingobacterium multivorum* w15, spread over 92 contigs, had a length of 6,678,278 bp, with an average G + C content of 39.7%. Of the 6,087 annotated genes, 5,999 were predicted to encode proteins, of which 734 were detected as hypothetical proteins. There were 76 genes for RNAs, of which 10 rRNAs (eight 5S rRNA, one 23S rRNA, and one 16S rRNA) and 66 tRNAs. Three CRISPR repeat sequences were found. No plasmids were found (Table 1).

### Taxonomic Affiliations

In previous work, the (partial) PCR-amplified rRNA gene sequence of *C. freundii* so4 showed 99% similarity with that of the type strain *C. freundii* DSM 30039^T^ (Cortes-Tolalpa et al., 2016), which formed the basis for its assignment to the species *C. freundii*. Here, we extend this analysis by aligning the full 16S rRNA gene found in the genome with that of closely related strains, including the type strain. The phylogenetic tree indeed confirms strain so4 to have a very close phylogenetic relationship (>99% match with the full sequence) with strains assigned to *Citrobacter freundii*, including the type strain DSM 30039^T^ (Supplementary Figure 1A). Consistent with this, it showed to be mesophilic, growing optimally on Lennox medium at around 30°C (Supplementary Figure 5A).

In a similar way, we extend the previous analysis of *S. multivorum* w15 based on the PCR-amplified 16S rRNA gene sequence to the full 16S rRNA gene sequence from the genome. The sequence clustered into a group of sequences obtained from diverse organisms that were all classified as *S. multivorum* (or alike); it was 99% similar to the sequence of the type strain *S. multivorum* IAM 14316^T^. In the phylogenetic tree, *S. multivorum* w15 is presented in bold (Supplementary Figure 1B). *S. multivorum* w15 revealed optimal growth at 28°C, hence was also mesophilic (Supplementary Figure 5B).

### Assignment of Translated Genes to COG Categories

The genome of *C. freundii* so4 was found to have 3,915 protein-encoding genes that match COG functional categories, representing 83.25% of the total and leaving 16.75% of the genes unclassified. Among the COG-recognized genes, several hundreds were found to be related with energy production and conversion (307), amino acid transport and metabolism (480) and carbohydrate transport and metabolism (413) (Table 2).

**TABLE 2:** Number of genes associated with general COG functional categories.

	***C. freundii* so4**		***S. multivorum* w15**		
**Code**	**Value**	**% of total^a^**	**Value**	**% of total^a^**	**Description**
J	190	4.15	188	3.13	Translation, ribosomal structure and biogenesis
K	375	8.20	460	7.66	RNA processing and modification
A	1	0.02	–	–	Transcription
L	174	3.81	204	3.40	Replication, recombination and repair
B	–	–	1	0.02	Chromatin structure and dynamics
D	47	1.03	40	0.66	Cell cycle control, Cell division, chromosome partitioning
V	49	1.07	105	1.75	Defense mechanisms
T	218	4.77	312	5.20	Signal transduction mechanisms
M	259	5.67	333	5.55	Cell wall/membrane biogenesis
N	130	2.85	20	0.33	Cell motility
U	123	2.69	85	1.42	Intracellular trafficking and secretion
O	149	3.26	182	3.03	Posttranslational modification, protein turnover, chaperones
C	307	6.72	225	3.75	Energy production and conversion
G	413	9.04	355	5.91	Carbohydrate transport and metabolism
E	480	10.51	341	5.68	Amino acid transport and metabolism
F	85	1.86	73	1.22	Nucleotide transport and metabolism
H	165	3.61	147	2.45	Coenzyme transport and metabolism
I	134	2.93	147	2.45	Lipid transport and metabolism
P	345	7.55	425	7.08	Inorganic ion transport and metabolism
Q	111	2.43	90	1.50	Secondary metabolites biosynthesis, transport and catabolism
R	559	12.24	638	10.62	General function prediction only
S	346	7.57	326	5.43	Function unknown

*^*a*^Based on the total number of protein-encoding genes in the genome; –, not detected.*

In contrast, only 63.31% of the protein-encoding genes of *S. multivorum* w15 (total: 3854) were associated with COG functional categories whereas the remaining 36.69% genes remained unclassified. Similar to *C. freundii* so4, hundreds of COG-defined genes were associated with energy production and conversion (225), amino acid transport and metabolism (341) and carbohydrate transport and metabolism (355).

On the basis of the above numerical data, strain w15 did not show big differences in its allocation of genetic resources as compared to strain so4. There was one conspicuous difference: the numbers of distinct genes associated with proteins involved in ‘defense’ (105) and ‘signal transduction’ (312) in the strain w15 genome strongly exceeded those in the strain so4 genome (respectively 49 and 218), both in absolute numbers and percentages (Table 2).

### General Metabolism

#### *C. freundii* so4

**FIGURE 2:**
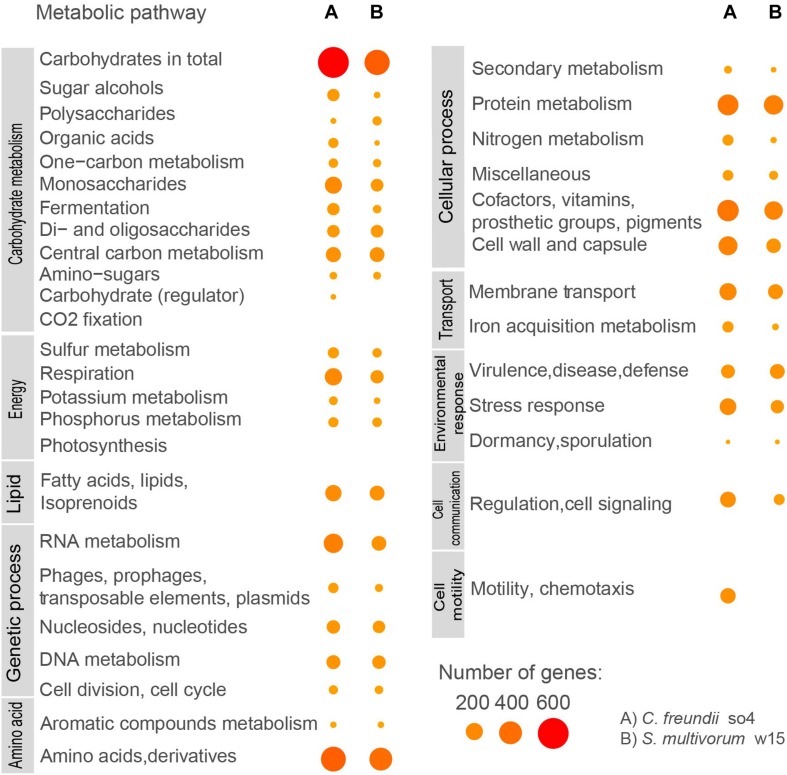
Predicted functional subsystems in *C. freundii* so4 and *S. multivorum* w15, based on RAST results and KEGG assignments. Sizes and colors of circles indicate numbers of genes assigned to each function.

Based on the RAST results (Supplementary Table 4), the most dominant functional category (that is, having the highest number of genes encoding proteins) in the *C. freundii* so4 genome was carbohydrate metabolism (706 genes, or 15.50% of the total). The second most abundant category was amino acid metabolism (438 genes), followed by cofactors/vitamins (314), protein metabolism (295), RNA metabolism (248), cell wall (236), respiration (188), membrane transport (187), stress responses (175) and lipid metabolism (166). Furthermore, *C. freundii* so4 revealed 143 genes associated with chemotaxis and motility (Figure 2), showing that *C. freundii* so4 can actively move in the system; this was experimentally proven, as shown in Supplementary Figure 2.

#### *S. multivorum* w15

With respect to *S. multivorum* w15, a total of 451 genes (7.52% of the genome) was found to be associated with carbohydrate metabolism. In order of decreasing abundance, genes related with amino acid metabolism (364), protein metabolism (250), cofactors and vitamins (222), membrane transport (134), defense systems (132), lipid metabolism (132), RNA metabolism (129), cell wall (125), and DNA metabolism (104) were found. Interestingly, *S. multivorum* w15 lacked genes associated with chemotaxis and motility; tests for motility and/or chemotaxis yielded negative results (Figure 2, Supplementary Table 4, and Supplementary Figure 2).

### Carbohydrate Degradation

**FIGURE 3:**
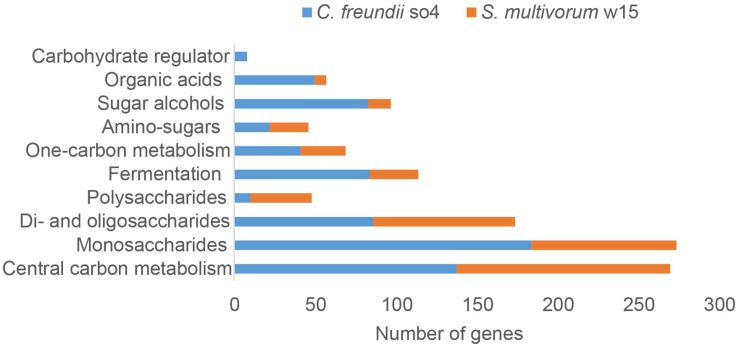
Numbers of genes encoding proteins associated with carbohydrate metabolism found in the genomes of *C. freundii* so4 (blue) and *S. multivorum* w15 (orange).

In both *C. freundii* so4 and *S. multivorum* w15, the majority of genes encoding carbohydrate-degradative proteins was associated with the transformation of mono- next to di- and oligo-saccharides (bottom of Figure 3). In *C. freundii* so4, high numbers of genes were involved in monosaccharide metabolism (184), central carbon metabolism (138), di- and oligo-saccharide metabolism (86), fermentation (84), sugar alcohol metabolism (83) and 1-carbon metabolism (41) (Figure 3). In contrast, *S. multivorum* w15 had two- to three-fold lower numbers of genes for carbohydrate-active proteins related to monosaccharide, one-carbon and fermentation metabolisms. The key ones were involved in: central carbon metabolism (132) transformation/utilization of monosaccharides (90) and of di- and oligo-saccharides (88), and fermentation (30). Even though the numbers of genes for sugar alcohol and organic acid metabolisms were not the highest in *C. freundii* so4 (83 and 50, respectively), these numbers were still far higher than those in *S. multivorum* w15 (14 and 7, respectively).

In contrast, the *S. multivorum* w15 genome contained 38 genes encoding proteins predicted to degrade polysaccharides, whereas the genome of *C. freundii* so4 had only 10. Here, the larger genomic complement found in *S. multivorum* w15 indicated that it is likely to be a vital player in the bipartite lignocellulose degradation process.

Interestingly, *C. freundii* so4 revealed a suite of genes (8) associated with regulation, i.e., ‘carbohydrate regulation,’ whereas such genes were not found in *S. multivorum* w15. Specifically, one gene, *csrA*, was predicted to encode a carbon storage regulator. The remaining seven genes were all located in the ‘carbohydrate utilization cluster’ denoted Ydj, encoding: a hypothetical aldolase (YdjI), an uncharacterized sugar kinase (YdjH), a hypothetical zinc-type alcohol dehydrogenase-like protein (YdjJ), a putative oxidoreductase (YdjL), a putative transport protein (YdjK), a hypothetical oxidoreductase (YdjG) and a putative HTH-type transcriptional regulator (YdjF). With respect to genes for di-saccharide (88) and amino sugar (24) metabolisms, *S. multivorum* w15 had similar gene numbers as *C. freundii* so4 (Figure 3).

### Analysis of Lignocellulolytic Potential

The genomes of both *C. freundii* so4 and *S. multivorum* w15 showed a plethora of genes predicted to encode proteins from several CAZy families and CBMs. There were important differences between the two genomes in the total numbers of genes associated with lignocellulose degradation.

#### GH and CBM Families

**FIGURE 4:**
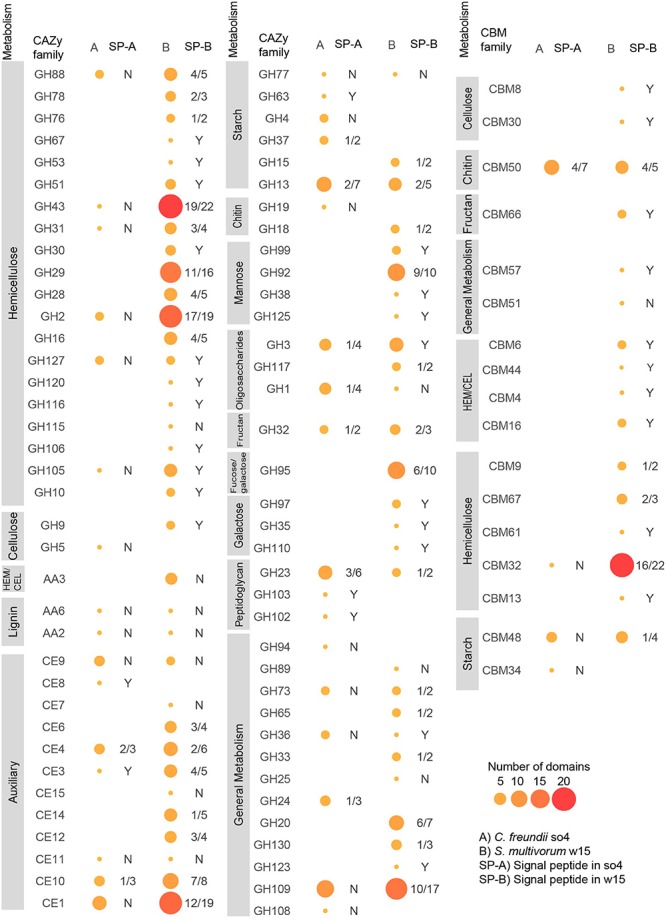
Domain distributions in predicted proteins matching different CAZy and CBM families in *C. freundii* so4 (A) and *S. multivorum* w15 (B). Glycosyl hydrolases (GH), carbohydrate binding modules (CBM), auxiliary activity (AA), carbohydrate esterases (CE). HEM/CEL: enzymes active on cellulose and hemicellulose. SP-A/B is the rate of occurrence of signal peptides in the proteins found in each strain: Y = 100%, N = 0%, 4/5 means 4 out of 5 domains identified in this enzyme family have signal peptides. Sizes and colors of circles indicate the number of domains associated with each family.

The genome of *C. freundii* so4 exhibited 130 genes having regions predicted to encode proteins associated with CAZy families and CBMs (Supplementary Figure 3); 125 of these 130 proteins had single (CAZy or CBM) domains, and 5 (3.85%) had multiple domains (Supplementary Table 5). Overall, 137 domains were identified: 61 GHs, 43 glycosyl hydrolases (GTs), 18 carbohydrate esterases (CEs), 12 CBMs, 2 auxiliary activities (AAs), and 1 polysaccharide lyases (PL) (Supplementary Figure 4). Among the five multi-domain proteins, two contained a single GH domain associated with one CBM (CBM34-GH13, CBM48-GH13), and two other ones contained a single GH domain associated with two (similar) CBMs (CBM48-CBM48-GH13, CBM50-CBM50-GH23). Finally, one protein contained a single GH domain and a GT domain (GT84-GH94) (Supplementary Table 6). Both the CBM48 and CBM34 modules are associated with attachment to starch; the latter was exclusively present in this strain. Specifically, out of the predicted 12 proteins with CBM domains, six were single-domain proteins, being five CBM50-like (chitin-binding) and one CBM32-like (Figure 4).

The genome of *S. multivorum* w15 exhibited 348 genes having regions predicted to encode proteins associated with CAZy families and CBMs, most of which could be directly linked with lignocellulose degradation (Supplementary Figure 3). Three hundred and thirteen out of the 348 proteins had single domains, and 35 (10.06%) multiple domains (Supplementary Table 5). Thus, 386 domains were identified across the board: 193 GHs, 65 GTs, 56 CEs, 50 CBMs, 6 AAs, and 12 PLs (Supplementary Figure 4). Among the 35 multi-domain proteins, 23 contained a single GH domain associated with one CBM (e.g., GH16-CBM16), and two contained one GH domain associated with two (similar) CBMs (GH29-CBM32-CBM32, GH23-CBM50-CBM50). Moreover, 6 proteins contained 2 GH domains (e.g., GH43-GH43, GH16-GH43), 1 contained 2 CBMs (CBM50-CBM50), 2 two CE domains (CE7-CE15, CE3-CE6) and a final one 3 different domains (CE4-GH18-GT2) (Supplementary Table 6). Specifically, out of the 50 predicted CBM domains, 21 were identified in single-domain proteins; CBM32 (12), CBM9 (2), CBM66 (2), CBM6 (1), CBM44 (1), CBM13 (1), CBM51 (1), and CBM8 (1).

Overall, the genome of strain w15 had genes encoding proteins from 48 different GH families and 16 different CBM families (Figure 4), whereas the genome of strain so4 had only 25 (GH) and 4 (CBM) such genes. When commonality was considered, we found that the two strains shared genes for proteins matching 36 CAZy families (Supplementary Figure 3). Conversely, regarding genes encoding proteins matching unique (CAZy and CBM) families, *S. multivorum* w15 had 62 unique types and *C. freundii* so4 20.

#### Genes Associated With Hemicellulose Degradation

The *C. freundii* so4 genome presented genes encoding proteins matching six GH families related to hemicellulose degradation, i.e., GH2, GH31, GH43, GH88, GH105, and GH127. These were also present in the genome of *S. multivorum* w15 (Figure 4).

The genome of *S. multivorum* w15 – uniquely – exhibited putative genes encoding proteins that matched 21 GH families involved in the degradation of hemicellulose. These were: GH2, GH10, GH16, GH28, GH29, GH30, GH31, GH43, GH51, GH53, GH67, GH76, GH78, GH88, GH92, GH105, GH106, GH115, GH116, GH120, and GH127 (Figure 4). The families with most domains identified were GH2 (19), GH29 (16), GH43 (22), and GH92 (10). Moreover, we found evidence for proteins with two different CBMs predicted to bind to xylan: CBM9 and CBM13 (all as single domain proteins). The genome also harbored genes codifying predicted proteins with CBMs capable of binding to cellulose, xylan, glucan and glucomannan, namely CBM4 (GH10-CBM4), CBM6 (CBM6-GH43), CBM16 (CBM16-GH16) and CBM44 (identified in a single domain protein). Remarkably, proteins with CBM4 type domains are predicted to bind to crystalline cellulose, a very recalcitrant part of the lignocellulose substrate (Figure 4). Surprisingly, *S. multivorum* w15 showed evidence for the production of 22 proteins with family CBM32 domains (associated with GH29, GH43, GH31, GH2, GH20) and three of family CBM67 (associated to GH35 and GH78), as well as CBM48 (GH13-CBM48) (binding to starch), CBM50 (associated to GH23, GH73) (chitin), CBM66 (identified in a single domain protein) (fructan) and CBM61 (GH43-CBM61) (galactan) (Supplementary Table 6).

#### Genes Associated With Cellulose Degradation

Overall, *S. multivorum* w15 had more putative genes involved in cellulose degradation than *C. freundii* so4, as shown in Figure 4. Thus, the *C. freundii* so4 genome had only one putative gene encoding a protein from CAZy family GH5, which may be associated with the degradation of crystalline cellulose (Figure 4).

The genome of *S. multivorum* w15 uniquely presented putative genes encoding enzymes matching CAZy family GH9 (associated with cellulose degradation). Moreover, the *S. multivorum* w15 genome exclusively revealed the presence of predicted genes encoding proteins with domains from CBM8 and CBM30 families, which are associated with binding to cellulose.

#### Genes for Auxiliary Enzymes

Genes encoding enzymes of seven CE families [CE1 (6), CE3 (1), CE4 (3), CE8 (1), CE9 (3), CE10 (3) and CE11 (1)] were found in the genome of *C. freundii* so4 (Figure 4). In contrast, the genome of *S. multivorum* w15 revealed the presence of ample genes encoding enzymes of eleven CE families, including families CE1 (19), CE3 (5), CE4 (6), CE9 (2) CE10 (8), and CE11 (1). It uniquely had genes encoding proteins of the remaining five CE families, i.e., CE6, CE7, CE12, CE14, and CE15. Members of these protein groups have been associated with deacetylation of xylans and xylo-oligosaccharides. Also, family CE15 proteins may be responsible for breaking recalcitrant links between hemicellulose and lignin. The *S. multivorum* w15 genome further had four genes encoding proteins of family AA3, which includes enzymatic activities of cellobiose dehydrogenase, dehydrogenase, glucose oxidoreductases, aryl-alcohol oxidase, alcohol (methanol) oxidase, and pyranose oxidoreductases. These enzymes support the action of glycoside hydrolases in lignocellulose degradation and protein structural analysis indicated that such enzymes may degrade and modify cellulose, hemicellulose and even lignin (Sützl et al., 2018).

#### Genes for Other CAZy Family Proteins

The genome of *C. freundii* so4 uniquely revealed genes encoding enzymes from three CAZy families associated with starch degradation, i.e., GH4, GH37, and GH63. The genome also exhibited genes for family GH13 and GH77 enzymes, which is shared with the *S. multivorum* w15 genome (Figure 4). *S. multivorum* w15 uniquely exhibited the presence of genes encoding CAZy family GH15 proteins (related to starch degradation). Moreover, it revealed a gene encoding a family GH110 protein, a polysaccharide depolymerase, which can hydrolyze galactosyl-alpha-1, 3-D-galactose linkages that are typically present in complex substrates (Figure 4).

In both genomes, we also found genes for family AA2 peroxidases, at one copy each, which are predicted to be involved in lignin degradation. Members of lytic cellulose mono oxygenases (AA10 family) were not found in any of the two genomes. Genes for CAZy family GH36 proteins were found in both genomes; the family encompasses enzymes that degrade the oligosaccharides stachyose and raffinose, present in a wide variety of plants (CAZypedia Consortium, 2017). However, *C. freundii* so4 only grew on raffinose as a single carbon source, whereas *S. multivorum* w15 could survive on both stachyose and raffinose as the single carbon source (Supplementary Tables 2, 3).

#### CAZy and CBM Family Enzymes – Secreted or Intracellular?

In 23 of the 130 predicted proteins matching CAZy families or CBMs of *C. freundii* so4, signal peptides were found (Figure 4), indicating these might be extracellular. Thus, about 18% of these proteins were secreted versus 82% potentially intracellular. Four of these were predicted to target starch, five peptidoglycan, four chitin, five ‘carbohydrates’ (esterases), three oligosaccharides, one fructan and one related to glycosyl transfer.

Of the 348 *S. multivorum* w15 predicted proteins matching CAZY families or CBMs, 202 had signal peptides (Figure 4). So, >58% of these proteins were secreted versus <42% intracellular. Among the former, 93 were predicted to target hemicellulose, 31 ‘carbohydrates’ (esterases), three cellulose, 13 mannose, 6 fucose and galactose, 2 active on both hemicellulose and cellulose, 3 starch, 10 ‘polysaccharides’ (lyases), 7 oligosaccharides, 4 fructan, 3 galactose and 3 chitin. Next to this, two had dockerins, 21 were ‘general metabolism’, and one a glycosyltransferase.

## Discussion

*Citrobacter freundii* so4 and *S. multivorum* w15 form part of a core set of bacteria that are highly abundant in LCB degrader consortia, indicating their key roles in lignocellulose degradation (Jiménez et al., 2014a; Brossi de Lima et al., 2015; Cortes-Tolalpa et al., 2016). Both strains can grow alone on WS as the sole carbon source. However, when growing together on WS, they were found to present a tight synergistic relationship (Cortes-Tolalpa et al., 2017). Clearly, knowledge of both the metabolic potential and the genomic features of the two bacteria will advance our understanding of the mechanisms behind this synergism. The data presented here show that metabolic differences and diverse polysaccharide degradation armories are potentially at the basis of the cooperation. When growing together on LCB, strain so4 and w15 may use their degrader metabolic capacities in a complementary fashion, allowing them to consume the substrates in a more efficient way than either one of them alone. Hereunder, we explore the differences found in the metabolic palettes of the two strains.

### Proposed Complementary Roles of *C. freundii* so4 and *S. multivorum* w15 in Wheat Straw Degradation

Our analyses indicated that differences in catabolism between *S. multivorum* w15 and *C. freundii* so4 growing on WS may overwhelm the competition for the same nutritional source (Figure 2 and Table 3). Overall, the genomic data and the carbon consumption profiles indicated that *C. freundii* so4 had a metabolism that is tuned toward the transformation of simple carbon sources, such as amino acids and metabolic intermediates of glycolysis and the TCA, using these pathways for the generation of energy (Figure 1 and Table 2). Interestingly, the organism revealed enzymatic capacities for pyruvate, propanoate and ascorbate-aldarate metabolisms; the predicted proteins might be main players in (facultatively) anaerobic metabolisms (Table 3). Further predictive analyses showed that strain so4 had an active glutathione pathway (Table 3), which is capable of preventing damage to key cellular components caused by reactive oxygen species, such as in the detoxification of formaldehydes. Moreover, a capacity of mixed acid fermentation was present, which allows *C. freundii* so4 to grow under limited oxygen conditions (Figure 1 and Supplementary Table 1). In contrast, *S. multivorum* w15 (which is a strictly aerobic organism), presented a strong preference for consumption of more complex carbohydrates (Figure 1 and Supplementary Table 2). The organism probably makes use of the pentose interconversion pathway, as it appears to have considerably diminished its investment in the pyruvate, ascorbate and propionate pathways. *S. multivorum* w15 potentially is a prime degrader of WS polymers, making the WS more accessible to strain so4.

**TABLE 3:** Number of distinctive enzymes observed in different metabolic pathways found in *C. freundii* so4 and *S. multivorum* w15.

**Pathway**	**Distinct ECs**	***C. freundii* so4 (%)**	***S. multivorum* w15 (%)**
Citrate cycle (TCA cycle)	41	63	59
Glycolysis/Gluconeogenesis	22	58	49
Pentose phosphate pathway	37	62	57
Pyruvate metabolism	64	51	37
Propionate metabolism	47	44	21
Starch and sucrose metabolism	71	30	30
Pentose and glucuronate interconversions	56	45	39
Inositol phosphate metabolism	40	22	15
ß-Alanine metabolism	32	28	19
Ascorbate and aldarate metabolism	44	39	25
Glutathione metabolism	40	42	25

*Values represent the percentages of genes/enzymes needed for a functional pathway. Reference metabolic pathway highlighted (KEGG data based). Distinct reactions per metabolic pathway according to the enzyme commission (EC) number. Comparison based on KEGG database. EC numbers do not specify enzymes, but enzyme-catalyzed reactions. If different enzymes, for instance from different organisms, catalyze the same reaction, then they receive the same EC number.*

With respect to lignocellulose degradation, *C. freundii* so4 apparently prefers to consume (intermediary) sugars, products of cellulose hydrolysis and disaccharides with beta-glycosidic bonds, such as cellobiose (glucose β (1→4) glucose) and galactose (β-D-galactosepyranosyl-D-glucopyranose) (Figure 1 and Supplementary Table 1). In contrast, *S. multivorum* w15 shows a propensity to utilize carbohydrates with α bonds such as glucose α (1→4) glucose, melibiose (D-gal- α 1→6 D-glucose) and γ-cyclodextrin (Figure 1 and Supplementary Table 2).

Another example of how the strains may complement each other with respect to their metabolism is the following. *C. freundii* so4 is highly versatile in its capacity to spatially explore a substrate like WS, as it, due to its flagellar apparatus, can swim to explore the locally available resources, whereas *S. multivorum* w15 cannot. This forces the latter organism to produce the plethora of extracellular enzymes that are locally required for the digestion of substrate, thus acquiring the resulting smaller molecules. It is possible that *C. freundii* so4 – given its ability to move around - can readily reach those sites at the substrate where nutrients become available, taking these up in a motility-facilitated manner.

### Model – Depiction of Roles of the Strains in the Wheat Straw Degradation System

**FIGURE 5:**
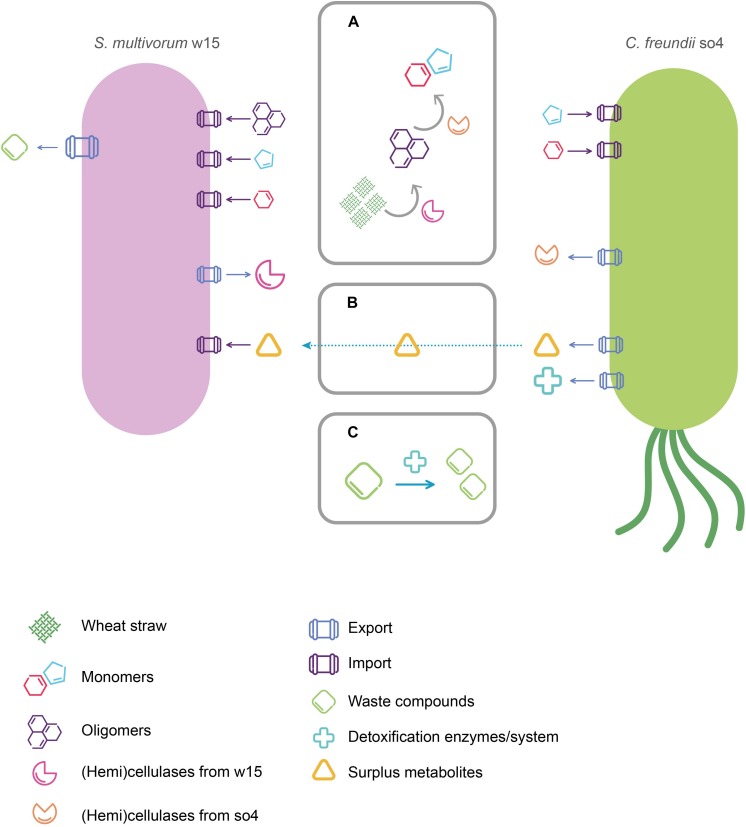
Proposed roles of *C. freundii* so4 and *S. multivorum* w15 in the degradation of wheat straw, and mode of interaction. **(A)** Attack on wheat straw by (hemi)cellulases produced by *C. freundii* so4 and *S. multivorum* w15 (attacks on other compounds not depicted). **(B)** Potential metabolites of *C. freundii* so4 captured by *S. multivorum* w15. **(C)** Waste/toxic compounds removed by *C. freundii* so4.

Cooperation based on metabolic exchange occurs when a species uses metabolites produced by another species as sources of energy or building blocks for cell structures (Cavaliere et al., 2017). The process is also known by the term cross-feeding. A key example is given by one strain degrading a primary carbon and energy source and producing a compound that is then used by a second strain (Germerodt et al., 2016). We propose that the two strains studied here, *C. freundii* so4 and *S. multivorum* w15, exhibit a cooperative cross-feeding interaction (Figure 5). While *S. multivorum* w15 primarily invests in the degradation of the WS hemicellulose, *C. freundii* so4 has other functions in the system, e.g., transforming oligo-intermediates. The consumption of the latter allows strain so4 to grow, and eventually produce secondary metabolites, that *S. multivorum* w15 can use. Also, *C. freundii* so4 could contribute to the detoxification of compounds in the culture (Figure 5). Three hypotheses might thus explain the positive relationship between the two strains as found on WS: (1) complementary degradation capacity, (2) production and excretion of secondary metabolites, and (3) stress response based mutualism.

#### Complementary Degradation Capacities

We posit here that *S. multivorum* w15 serves as the primary degrader of a complex substrate like WS, contributing with the production and release of a large variety of hydrolytic enzymes. Clearly, large parts of its genome are tuned to the degradation of xylan with different crosslinks, which is a main component of hemicellulose. As evidenced on the basis of the genome analyses, *S. multivorum* w15 may use mainly proteins of CAZy families GH29 and GH43. The family GH29 proteins may be exo-acting α-fucosidases, which participate in glycan degradation (CAZypedia Consortium, 2017). In addition, the main functions reported for CAZy family GH43 enzymes are α-L-arabinofuranosidases, endo-α-L-arabinanases, β-D-xylosidases and galactosidases. A large number of enzymes in this family shows dual (α-L-arabinofuranosidase and β-D-xylosidase) activities, using aryl-glycosides as substrates (CAZypedia Consortium, 2017). For instance, Maruthamuthu et al. (2017) reported that a novel GH43 family enzyme had β-xylosidase/α-arabinosidase activities. Family GH43 enzymes are clearly implicated in the degradation of arabinoxylan, the most abundant hemicellulose component of WS (Abot et al., 2016; Mewis et al., 2016). *S. multivorum* w15 may also employ enzymes from CAZy family GH2, which encompasses β-galactosidases, β-glucuronidases, β-mannosidases, and exo-β-glucosaminidases. Moreover, the finding of genes for CBM32-type proteins may indicate a capacity of taking up monosaccharides and short oligosaccharides (CAZypedia Consortium, 2017). Furthermore, the finding of genes for carbohydrate esterase family 1 (CE1) proteins was revealing. Thus, *S. multivorum* w15 may accelerate the degradation of polysaccharides, facilitating the access of glycoside hydrolases to the substrate (Nakamura et al., 2017). CE1 is one of the largest and most diverse CE families, including acetyl xylan esterases, feruloyl esterases, and carboxyl esterases that carry out the deacetylation of xylan and oligosaccharides.

On the other hand, the current evidence points to a role for *C. freundii* so4 as a *consumer* of smaller carbonaceous molecules, transforming such substrate fragments, which are produced by the action of *S. multivorum* w15, into simpler ones on the way to CO_2_. What then is the role of this organism as a contributor to the complex substrate degradation? *C. freundii* so4 may provide lytic enzymes (GH5 family) that are different from those of *S. multivorum* w15 (GH9 family). The most common enzymes in family GH1 are β-glucosidases and β-galactosidases, next to β-mannosidases, β-D-fucosidases and β-glucuronidases (CAZypedia Consortium, 2017). Furthermore, others belong to family GH13, which is the major glycoside hydrolase family acting on substrates containing α-glucoside linkages (hydrolases, transglycosidases and isomerases – CAZypedia Consortium, 2017).

*Citrobacter freundii* so4 may also contribute to the WS degradation process with extracellular cellobiohydrolases that transform cellobiose into glucose monomers, which both strains can easily consume. Thus, it may hamper potential feedback inhibition processes as further discussed below. In cross feeding interactions, intermediate (by-)products of the degradation may inhibit the process (Harvey et al., 2014). An intriguing hypothesis is thus that *C. freundii* so4 contributes to the system by processing intermediates of cellulose degradation, such as cellobiose, as degradation may be inhibited by accumulation of product. Thus, by reducing the levels of (sugar) products of *S. multivorum*’s lytic activity, *C. freundii* so4 may promote the activity of such enzymes in the biculture.

#### Production and Excretion of Metabolites

Although speculative, *C. freundii* so4 may also contribute to the degradative system by producing and excreting metabolites that *S. multivorum* w15 can use, but cannot produce (for example amino acids and derivatives). Such metabolites that (temporarily) cannot be transformed, may be required to be transported out of the cell.

#### Stress Response Modulation

The degradation of WS by the two strains may produce potentially toxic intermediary metabolites that accumulate in the medium. These can reduce growth and enzyme production. For instance, phenolic compounds, aldehydes and furan derivatives, next to oxidative stress compounds, are known to exert this action (Malherbe and Cloete, 2002; Ling et al., 2014). Our genome analysis revealed, particularly in *C. freundii* so4 (but not in *S. multivorum* w15), the presence of:

(1) A regulon containing the oxidative stress response regulators SoxS and SoxR,
(2) Genes associated with glutathione metabolism and glutathione transcriptional regulator (formaldehyde detoxification operon – FrmR),
(3) Genes associated with nitrosative stress, specifically those for fumarate and nitrate reduction regulatory proteins,
(4) Diverse oxidoreductases that may detoxify xenobiotics, for instance, phenolics, oxidizing inorganic compounds using oxygen as the final electron acceptor (Karigar and Rao, 2011).

The finding of these genes in *C. freundii* so4 but not in *S. multivorum* w15 is consistent with the tenet that strain so4 assists in detoxification and oxidative stress relief processes. Thus, levels of accumulated waste compounds may be reduced in the culture.

## Conclusion and Perspectives

The differences in the metabolic potential between *C. freundii* so4 and *S. multivorum* w15 make these two organisms complementary in WS degradation, as different roles for these in the system can be cogitated. Whereas *S. multivorum* w15 may have its main role in primary degradation, in particular releasing hemicellulose hydrolytic enzymes, *C. freundii* so4 may contribute with detoxification of the system, transforming sub-products of the degradation and providing intermediate metabolites that *S. multivorum* w15 cannot synthesize. In this way, both strains benefit from the joint activities, yielding improved growth on a very recalcitrant carbon source.

Our study provides a starting point for an improved understanding of cooperative degrader consortia. Importantly, key target genes, e.g., those encoding proteins from CAZy families GH2, GH29, GH43, GH109, CE1, CE3, CE4, CE14, and CE15, as well as CBM32, were found that await further analyses. Based on these data, we suggest focusing on the expression of genes encoding proteins with CBM32 domains and enzymes from family GH43. Evidence for the importance of proteins of the latter family (Jiménez et al., 2015; López-Mondéjar et al., 2016) has been provided uniquely when strains or consortia were grow on WS, xylan and xylose.

Moreover, enzymes involved in attacks on recalcitrant regions in lignocellulose substrate need to be studied, such as members of CAZy families CE3, CE4, CE14 and CE15. Only few studies have addressed these families of enzymes in bacteria, despite the fact that many bacterial species have genes encoding them, including homologs of fungal enzymes (De Santi et al., 2016).

At the metabolic level, it is necessary to confirm the participation of *C. freundii* so4 in the system by studying the expression of metabolic pathways for the synthesis of metabolites and elimination of toxic compounds. The knowledge generated from, for instance, transcriptome analyses can be used for modulation of the system and also in the synthesis of enzyme cocktails that include hydrolytic, debranching and auxiliary enzymes for lignocellulose treatment.

## Data Availability Statement

The datasets generated for this study can be found in the DDBJ/ENA/GenBank, PHGU00000000 and PHGV00000000.

## Author Contributions

LC-T, JS, and JE conceived this project. LC-T and YW analyzed the CAZy data, and wrote the manuscript, with comments and revisions by JS and JE. All authors read and approved the final manuscript.

## Conflict of Interest

LC-T is employed by the company Agilent Technologies.

The remaining authors declare that the research was conducted in the absence of any commercial or financial relationships that could be construed as a potential conflict of interest.

## Footnotes

1 http://www.cazy.org/

2 https://www.cazypedia.org/index.php/Main_Page
