# Supplementary material for: Comparative Genome Analysis of the Lignocellulose Degrading Bacteria Citrobacter freundii so4 and Sphingobacterium multivorum w15
Source: Front Microbiol. 2020 Mar 3;11:248. doi: 10.3389/fmicb.2020.00248 (PMC7065263; doi:10.3389/fmicb.2020.00248)
Supplement: Supplementary file 1 [file Data_Sheet_1.pdf]

Supplementary Material

1 Supplementary Data

1.1 Motility assay

Motility of *C. freundii* so4 and *S. multivorum* w15 was assessed by growing them on Motility Test Medium (10g/L pancreatin digest of casein, 3g/L NaCl, 4 g/L meat extract and 4 g/L agar) with triphenyltetrazolium chloride (TTC: 0.5 g/L, Sigma-Aldrich, Darmstadt, Germany). Tetrazolium salt is colorless; as the microorganism grows, it is reduced to an insoluble red pigment. Motile organisms produce a pink color that diffuses from the stab line. Organisms that are non-motile produce a red pigment that is confined to the stab line (Kelly and Fulton 1953).

1.2 Determination of temperature growth range

The temperature growth range of *C. freundii* so4 and *S. multivorum* w15 was determined by growing the strains on Lennox media (Sigma-Aldrich, Darmstadt, Germany) and monitoring growth at different temperatures (4, 20, 30, 40, 45, 50, 60 and 80 °C).

C. freundii so4 revealed to be mesophilic, as it was able to grow in the temperature range from 20°C to 30°C, not showing growth at 45°C, 60°C and 80°C (Supplementary Figures 5A), while the optimal temperature of growth was around 30°C.

S. multivorum w15 also showed a mesophilic temperature range of growth, from 20°C to 30°C, with low growth at 20°C; the optimal temperature of growth was 28 °C (Supplementary Figures 5B). It did not show growth at 40°C, 45°C, 60°C and 80°C.

2 Supplementary Figures and Tables

2.1 Supplementary Tables

Supplementary Table 1. Carbon sources used only by *C. freundii* so4

Compound	ID KEGG	Carbon source	Type
Putrescine	C00134	Amine	Ester
L-serine	C00065	Amino acid	Amino acid
D-serine	C00740	Amino acid	Amino acid
Hydroxy-L-proline	C01015	Amino acid	Amino acid
L-alanyl-glycine		Amino acid	Amino acid
L-proline	C00148	Amino acid	Amino acid
L-histidine	C00135	Amino acid	Amino acid
L-alanine	C00041	Amino acid	Amino acid
L-aspartic acid	C00049	Amino acid	Amino acid
D-glucosamine	C00329	Carbohydrate	Amino sugar
Dihydroxyacetone	C00184	Carbohydrate	Ketoses
Glycerol	C00116	Carbohydrate	Sugar alcohol
D-sorbitol	C00794	Carbohydrate	Sugar alcohol
D-mannitol	C00392	Carbohydrate	Sugar alcohol
m-inositol	C00137	Carbohydrate	Sugar alcohol
D-arabinose	C00216	Carbohydrate	Monosaccharide
Glucose-6-phosphate	C00092	Carbohydrate	Monosaccharide
L-fucose	C01019	Carbohydrate	Deoxy sugar

Succinic acid	C00042	Carboxylic acid	Carboxylic acid
5-Keto-D-gluconic acid	C01062	Carboxylic acid	Carboxylic acid
D-glucuronic acid	C00191	Carboxylic acid	Carboxylic acid
D,L-lactic acid	C01432(L)	Carboxylic acid	Carboxylic acid
D-galacturonic acid	C00333	Carboxylic acid	Acid sugar
D-gluconic acid	C00257	Carboxylic acid	Acid sugar
D-saccharic acid	C00818	Carboxylic acid	Acid sugar
Melibionic acid		Carboxylic acid	Acid sugar
Methylpyruvate		Ester	Ester
D-galactonic acid lactone	C03383	Ester	Ester
Inosine	C00294	Nucleic acid	Nucleoside
Thymidine	C00214	Nucleic acid	Nucleoside

ID KEGG: KEGG identifier

Highlighted compounds are related with lignocellulose degradation.

Supplementary Table 2. Carbon sources used only by *S. multivorum* w15

Compound	ID KEGG	Carbon source	Type
Glucuronamide	D01791	Amide	Amide
D-melezitose	C08243	Carbohydrate	Trisaccharide
Stachyose	C01613	Carbohydrate	Tetrasaccharide
Salicin	C01451	Carbohydrate	Monosaccharide
Lactulose	C07064	Carbohydrate	Disaccharide
Palatinose (Isomaltulose)	C01742	Carbohydrate	Disaccharide
Sucrose	C00089	Carbohydrate	Disaccharide
Turanose	G03588/C19636	Carbohydrate	Disaccharide
Gentiobiose	C08240	Carbohydrate	Disaccharide
α-Methyl-D-glucoside		Carbohydrate	Derived sugar
α-Methyl-D-mannoside		Carbohydrate	Derived sugar
Maltitol	G00275	Carbohydrate	Disaccharide
Arbutin	C06186	Carbohydrate	Derived sugar
β-Hydroxybutyric acid	C01089	Carboxylic acid	Carboxylic acid
Inulin	G04981	Polymer	Polysaccharide
Pectin	C00714/ G10591	Polymer	Polysaccharide

Dextrin	C00721	Polymer	Oligosaccharide
α-Cyclodextrin	C00973	Polymer	Oligosaccharide
β-Cyclodextrin	C13183	Polymer	Oligosaccharide
**γ**-Cyclodextrin	C13183	Polymer	Oligosaccharide

ID KEGG: KEGG identifier

Highlighted compounds are related with lignocellulose degradation.

Supplementary Table 3. Compounds consumed by both *S. multivorum* w15 and *C. freundii* so4

Compound	ID KEGG	Carbon source	Type
2-Aminoethanol	C00189	Alcohol	Organic acid
D-alanine	C00133	Amino acid	Amino acid
D-raffinose	C00492	Carbohydrate	Trisaccharide
Glucose-1-phosphate	C00103	Carbohydrate	Monosaccharide
β-Methyl-D-galactoside	C03619	Carbohydrate	Monosaccharide
α-D-glucose	C00267	Carbohydrate	Monosaccharide
D-fructose	C00095	Carbohydrate	Monosaccharide
N-Acetyl-D-glucosamine	C00140	Carbohydrate	Monosaccharide
D-mannose	C00159	Carbohydrate	Monosaccharide
D-galactose	C00124	Carbohydrate	Monosaccharide
L-arabinose	C00259	Carbohydrate	Monosaccharide
β-Methyl-D-glucose		Carbohydrate	Derived sugar
Maltose	C00208	Carbohydrate	Disaccharide
D-melibiose	C05402	Carbohydrate	Disaccharide
α-D-lactose	C00984	Carbohydrate	Disaccharide
D-trehalose	C01083	Carbohydrate	Disaccharide
D-cellobiose	C00185	Carbohydrate	Disaccharide
L-rhamnose	C00507	Carbohydrate	Deoxy sugar
N-acetyl-D-galactosamine	C01132	Carbohydrate	Amino sugar
N-acetyl-neuraminic acid	C00270	Carbohydrate	Amino sugar
Uridine	C00299	Nucleic acid	Nucleoside
Laminarin	C00771	Polymer	Polysaccharide

ID KEGG: KEGG identifier；

Highlighted compounds are related with lignocellulose degradation.

Supplementary Table 4. Number of genes in the functional subsystems according to RAST assignations.

**Function subsystems**	***C. freundii* so4**	***S. multivorum* w15**
Carbohydrates (total)	706	451
CO_2 fixation	0	0
Respiration	188	100
Sulfur metabolism	65	40
Phosphorus metabolism	50	43
Potassium metabolism	33	14
Photosynthesis	0	0
Fatty acids, lipids and isoprenoids	166	132
Phages, prophages, transposable elements, plasmids	53	26
Nucleosides and nucleotides	104	86
DNA metabolism	114	104
RNA metabolism	248	129
Cell division and cell cycle	38	31
Amino acids and derivatives	438	364
Metabolism of aromatic compounds	12	13
Secondary metabolism	24	8
Protein metabolism	295	250
Nitrogen metabolism	62	12
Miscellaneous	57	36
Cofactors, vitamins, prosthetic groups, pigments	314	222
Cell wall and capsule	236	125
Membrane transport	187	134
Iron acquisition and metabolism	65	15
Virulence, disease and defense	110	132
Stress response	175	101
Dormancy and sporulation	3	4
Regulation and cell signaling	152	61
Motility and chemotaxis	143	0

Supplementary Table 5. Number of predicted proteins with lignocellulolytic potential according to dbCAN annotation.

	***C. freundii* so4**		***S. multivorum* w15**	
	value	% of total	value	% of total
Proteins (total)	130	100	348	100
Domain (total)	137	--	386	--
Single-domain proteins	125	96.15	313	89.94
Multi-domain proteins	5	3.85	35	10.06

Supplementary Table 6. Multi-domain proteins of *S. multivorum* w15 and *C. freundii* so4 according to dbCAN annotation.

Multi-domain proteins of w15			
ID-w15	domains		
fig|6666666.255838.peg.1106	CBM67	GH78	
fig|6666666.255838.peg.1447	CBM30	GH9	
fig|6666666.255838.peg.1702	CBM48	GH13	
fig|6666666.255838.peg.1806	GH43	CBM61	
fig|6666666.255838.peg.1970	GH43	CBM32	
fig|6666666.255838.peg.1989	GH2	CBM32	
fig|6666666.255838.peg.1990	GH35	CBM67	
fig|6666666.255838.peg.2483	GH2	CBM32	
fig|6666666.255838.peg.2505	GH20	CBM32	
fig|6666666.255838.peg.3172	GH29	CBM32	
fig|6666666.255838.peg.3286	GH73	CBM50	
fig|6666666.255838.peg.3405	GH2	CBM57	
fig|6666666.255838.peg.3498	CBM48	GH13	
fig|6666666.255838.peg.3499	CBM48	GH13	
fig|6666666.255838.peg.4216	CBM67	GH78	
fig|6666666.255838.peg.4415	CBM48	GH13	
fig|6666666.255838.peg.4460	GH29	CBM32	
fig|6666666.255838.peg.451	GH43	CBM6	
fig|6666666.255838.peg.464	CBM4	GH10	
fig|6666666.255838.peg.4715	GH31	CBM32	
fig|6666666.255838.peg.484	GH43	CBM32	
fig|6666666.255838.peg.5647	GH16	CBM16	
fig|6666666.255838.peg.782	GH16	CBM16	
fig|6666666.255838.peg.1273	GH43	GH43	
fig|6666666.255838.peg.1862	GH16	GH43	
fig|6666666.255838.peg.2027	GH43	GH43	
fig|6666666.255838.peg.4267	GH43	GH43	
fig|6666666.255838.peg.447	GH10	GH43	
fig|6666666.255838.peg.5281	GH43	GH43	
fig|6666666.255838.peg.4656	CBM50	CBM50	
fig|6666666.255838.peg.5389	CE7	CE15	
fig|6666666.255838.peg.777	CE3	CE6	
fig|6666666.255838.peg.1335	GH29	CBM32	CBM32

fig|6666666.255838.peg.2158	GH23	CBM50	CBM50
fig|6666666.255838.peg.619	CE4	GH18	GT2

multi domain proteins of so4

ID-so4	domains		
fig|6666666.254466.peg.1562	CBM34	GH13	
fig|6666666.254466.peg.168	CBM48	CBM48	GH13
fig|6666666.254466.peg.169	CBM48	GH13	
fig|6666666.254466.peg.1747	CBM50	CBM50	GH23
fig|6666666.254466.peg.4042	GT84	GH94	

ID-w15: Gene identifier, strain w15; ID-so4: Gene identifier, strain so4.

2.2 Supplementary Figures

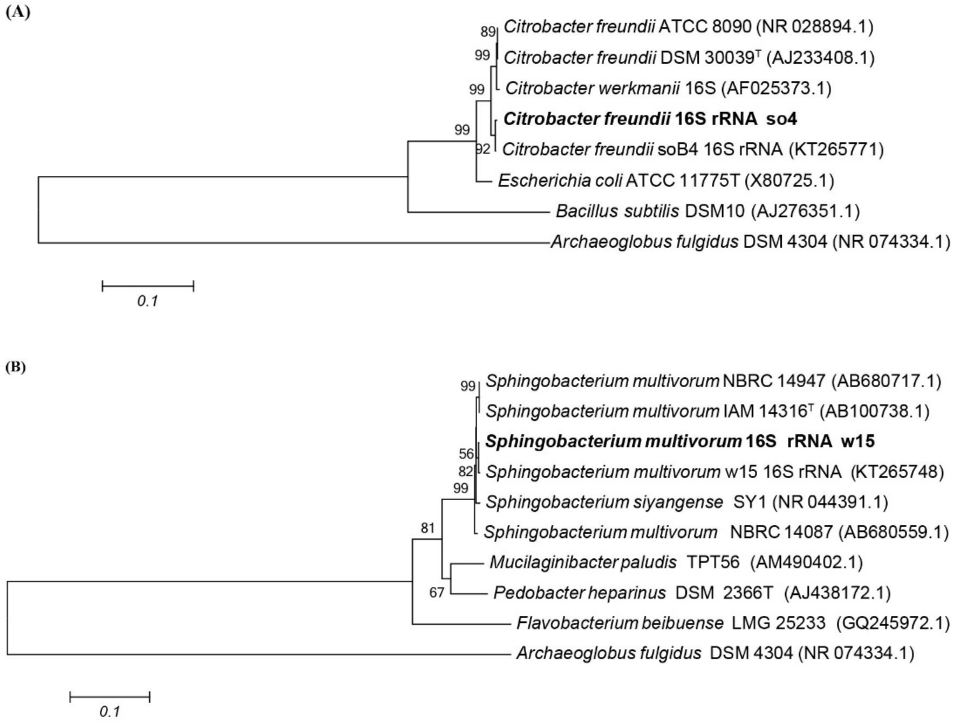

Supplementary Figure 1. Neighbour-joining phylogenetic tree based on 16S rRNA gene sequences. The tree indicates the relationship between (**A**) the isolated *C. freundii* so4 (**B**) the isolated *S. multivorum* w15, and other closely related strains, including the type strains. Isolates are shown in bold. Bootstrap values based on 1000 replications are listed as percentages at branching points. The sequence of *A. fulgidus* DSM 4304 was used as an out-group. Accession numbers are given in parentheses. The bars show 0.1 nucleotide substitutions per nucleotide position.

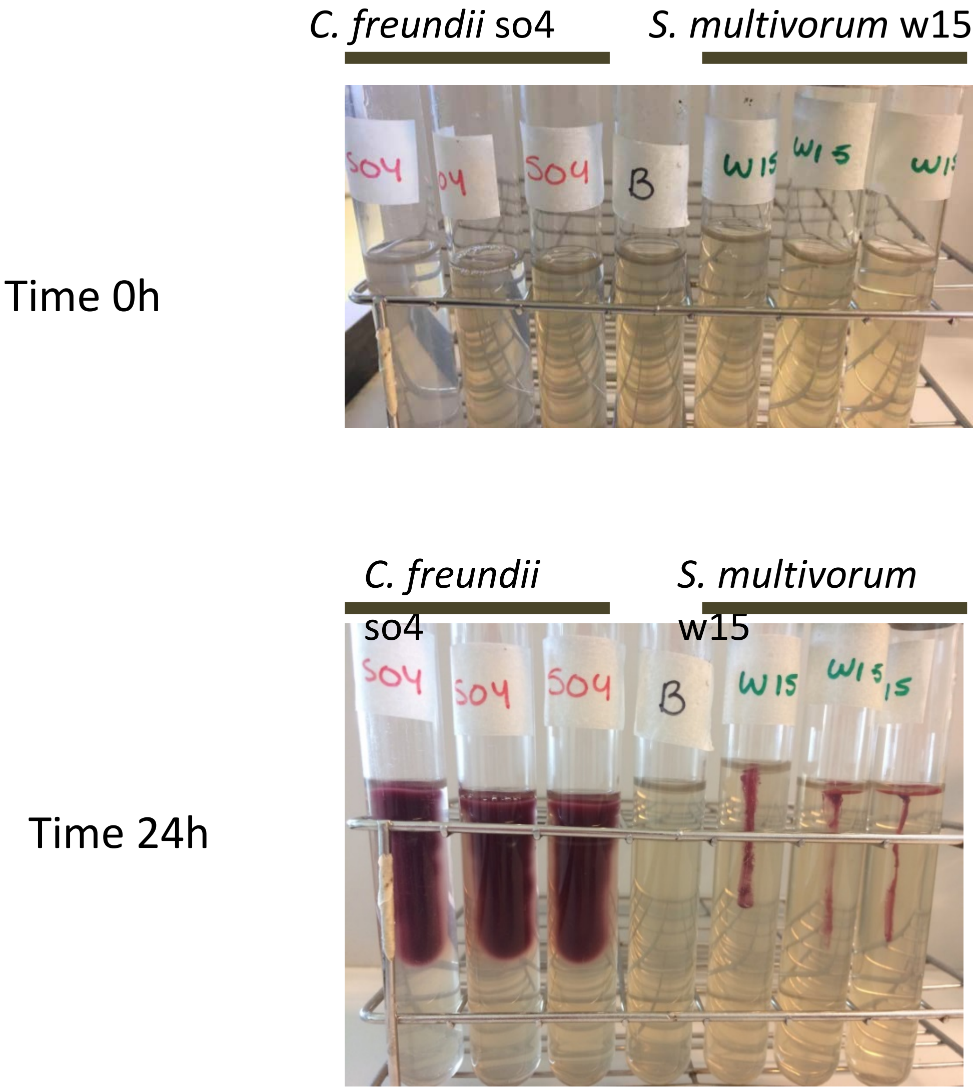

Supplementary Figure 2. Motility assay. *S. multivorum* w15 did not exhibite motility, while *C. freundii* so4 presented motility determined by the red coloration due to the oxidation of the triphenyltetrazolium chloride (TCC: 0.5 g/L). (A) Image shows inoculation point time; (B) Bacterial strains were incubated aerobically for 24 hours at 28ºC.

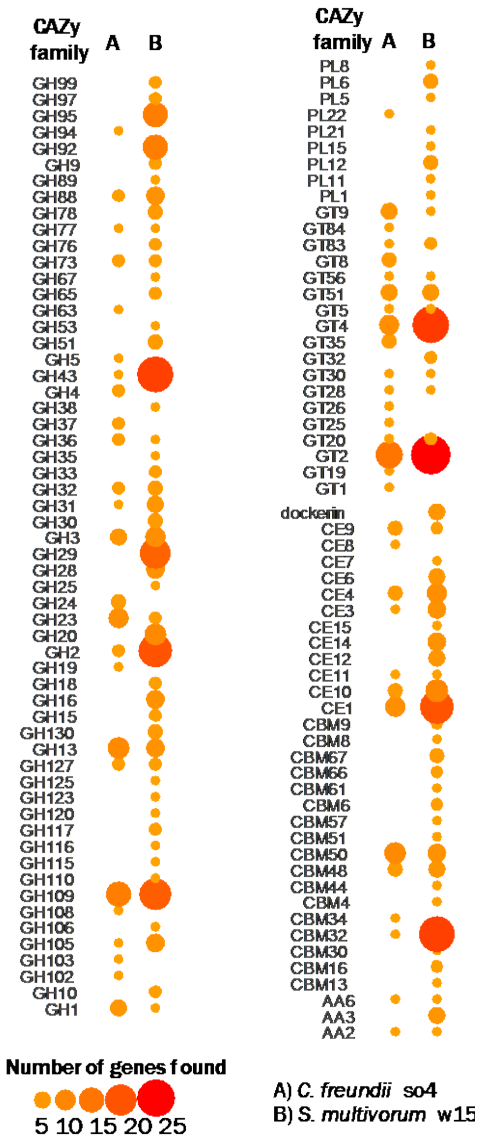

Supplementary Figure 3. Total genes predicted to encode proteins with domains matching CAZy families or CBMs in *C. freundii* so4 and *S. multivorum* w15. Sizes and colors of circles indicate predicted gene numbers. Glycosyl hydrolases (GH), carbohydrate binding modules (CBM), auxiliary activity enzymes (AA), polysaccharide lyases (PL), carbohydrate esterases (CE).

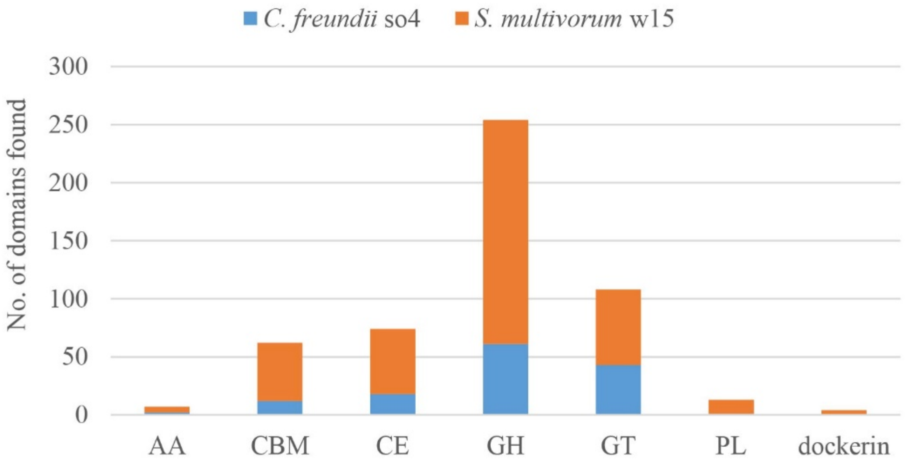

Supplementary Figure 4. Genes with domains matching CAZy family or CBM proteins found in the genomes of *C. freundii* so4 and *S. multivorum* w15. GH, glycosyl hydrolases; CBM, carbohydrate binding modules; AA, auxiliary activity enzymes; CE, carbohydrate esterases; GT, glycosyltransferases; PL, polysaccharide lyases.

(A) ***C. freundii*** **so4**

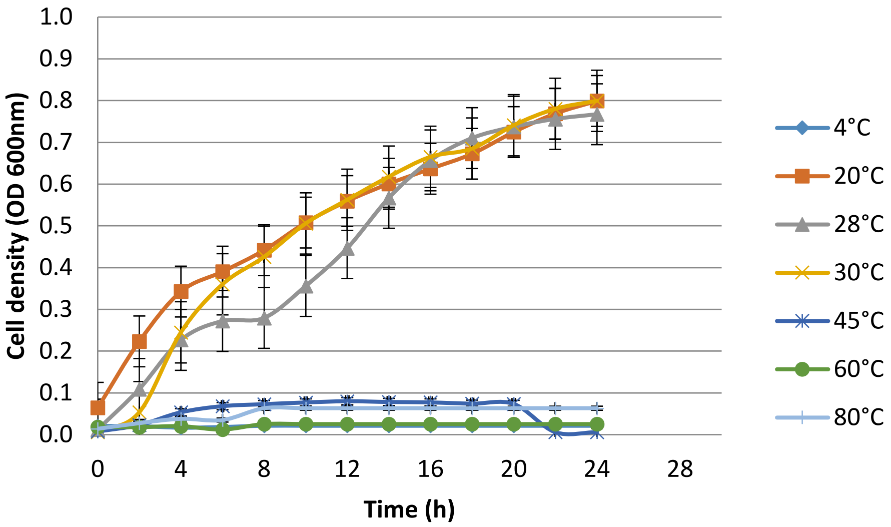

(B) ***S. multivorum*** **w15**

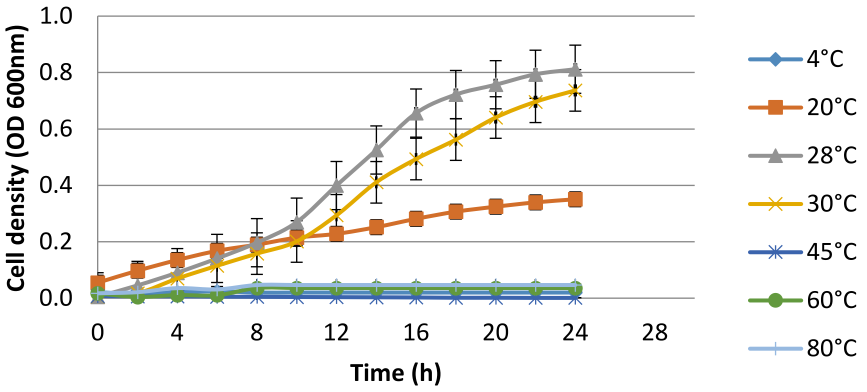

Supplementary Figure 5. Growth responses of (A) *C. freundii* so4 and (B) *S. multivorum* w15 at temperatures between 4 and 80℃.
